# The emergence of molecular systems neuroscience

**DOI:** 10.1186/s13041-021-00885-5

**Published:** 2022-01-04

**Authors:** Yang Shen, Alessandro Luchetti, Giselle Fernandes, Won Do Heo, Alcino J. Silva

**Affiliations:** 1 Departments of Neurobiology, Psychiatry and Biobehavioral Sciences, and Psychology, Integrative Center for Learning and Memory, and Brain Research Institute, UCLA, Los Angeles, CA USA; 2 Department of Biological Sciences, Korea Advanced Institute of Science and Technology, Daejeon, South Korea

**Keywords:** Systems neuroscience, Optogenetics, Molecular sensor and reporter, Imaging system

## Abstract

Systems neuroscience is focused on how ensemble properties in the brain, such as the activity of neuronal circuits, gives rise to internal brain states and behavior. Many of the studies in this field have traditionally involved electrophysiological recordings and computational approaches that attempt to decode how the brain transforms inputs into functional outputs. More recently, systems neuroscience has received an infusion of approaches and techniques that allow the manipulation (e.g., optogenetics, chemogenetics) and imaging (e.g., two-photon imaging, head mounted fluorescent microscopes) of neurons, neurocircuits, their inputs and outputs. Here, we will review novel approaches that allow the manipulation and imaging of specific molecular mechanisms in specific cells (not just neurons), cell ensembles and brain regions. These molecular approaches, with the specificity and temporal resolution appropriate for systems studies, promise to infuse the field with novel ideas, emphases and directions, and are motivating the emergence of a molecularly oriented systems neuroscience, a new discipline that studies how the spatial and temporal patterns of molecular systems modulate circuits and brain networks, and consequently shape the properties of brain states and behavior.

## Introduction

Systems neuroscience studies are focused on how particular circuits and large brain networks modulate brain states and behavior. Evolution has shaped circuits and brain networks to include a variety of cell types and a wide range of complex molecular properties. For example, within each cell of these networks, hundreds of thousands of molecules form complex systems that modulate the cellular responses that shape the output of these networks. Novel powerful techniques, that allow the tracking and manipulation of these molecular systems, in a cell specific manner and in real time, have fueled the emergence of molecular systems neuroscience, a sub-discipline of systems neuroscience that studies how the spatial and temporal patterns of molecular systems modulate circuits and brain networks, and consequently shape the properties of brain states and behavior. Molecular systems neuroscience studies attempt to explain brain processes by connecting molecular mechanisms to cellular and systems mechanisms in multiple brain regions engaged in processing information and generating behavior. Here, we will introduce this new field and review key examples of the exciting technological developments that motivated its emergence.

Systems neuroscience is traditionally focused on how the activity of neuronal circuits gives rise to internal brain states and behavior. Many of the studies in this field have typically involved recordings of action potentials and other electrophysiological properties in specific brain regions, such as the visual cortex and hippocampus of awake behaving animals. The principal goal of these studies has been to explain how a particular electrophysiological phenomenon accounted for the observed behavior. For example, the discovery in the hippocampus of neurons that fire in specific places (i.e., place fields) was compelling evidence for cognitive maps [1, 2]. In another example, the discovery of cells in the visual cortex that fired preferentially to simple stimuli, such as gradients of a particular angle, and other cells that preferred more complex stimuli, such as edges and movement, helped to explain how the visual system processes complex visual scenes from simpler stimuli within those scenes [3–6]. Another cornerstone of systems neuroscience has been analytical and theoretical approaches [7–9] that attempt to decode how the brain transforms inputs into functional outputs (e.g., how visual signals are used in the generation of visual perceptions and even cognitive maps). The computational tools in the field have deep roots in physics and engineering.

More recently, systems neuroscience has received an infusion of approaches and techniques that allow the manipulation (e.g., optogenetics [10–12], chemogenetics [13, 14]) and imaging (e.g., two-photon imaging [15–18], head mounted fluorescent microscopes [19–24]) of neurons, neurocircuits, their inputs and outputs in awake behaving animals. This exciting technological infusion has not only changed the questions asked, but even transformed the very epistemological basis of the field away from a reliance on the predictive power of theoretical approaches as the ultimate arbiter of ideas and hypotheses. Instead, the field is now relying increasingly more on the convergence of evidence and consistency of findings as the means to ground ideas and discoveries. For example, recent studies have been able to not only image neurons (e.g. activity and morphology) involved in a particular memory in real time as animals learn and remember (e.g., with GCAMP6f and head-mounted fluorescent microscopes), but also control the expression of the same memory by artificially activating or inhibiting those neurons through optogenetics and/or chemogenetics [25–27]. Similarly, studies have both imaged the neurons and manipulated the circuits involved in decision making, emotion, working memory, anxiety, vision, etc.

Novel approaches that allow the imaging and manipulation of specific molecular mechanisms in specific cells, cell ensembles and brain regions, promise to once again infuse systems neuroscience with novel ideas, emphases and directions. It is unlikely that all of the properties of systems and behavior will be reduced to molecular terms. Instead, we propose that the ability to image and manipulate specific molecular mechanisms in target cellular or subcellular sites, in specific circuits and brain regions, will help reveal unsuspected systems properties and functions.

There are three important challenges to traditional systems neuroscience that are driving the emergence of a more molecularly oriented version of the field. First, evolution molded the complex properties of multicellular systems by shaping over time the molecular properties of gene products. Thus, it is reasonable to propose that there are meaningful functional relations between genes, the proteins they encode, and (a) the processes that assemble circuits during development, and (b) the mechanisms that modulate their function in adult organisms. Inspired by evolution, insights on the function of genes in specific cells, cell systems and brain regions, may allow us to cut through the ever-growing complexity of how systems affect internal states and behavior.

Second, besides electrophysiological properties, other cell biological properties of neurons and other cells, such as mechanisms of transcription, translation, neuromodulation, receptor function, etc. also have a critical modulatory role in behavior. Indeed, there is growing evidence for the reasonable hypothesis that the orchestration of these mechanisms in neuronal ensembles is critical for internal brain states and behavior. Therefore, imaging and manipulating these molecular mechanisms has already and will continue to yield insights into how cellular systems control behavior.

Finally, there is now compelling evidence that the complex properties of behavior can be traced back to mechanisms that involve nearly every cell type in the brain, including, for example, astrocytes, oligodendrocytes and microglia. It is also reasonable to propose that the careful temporal and spatial organization of the myriad of molecular mechanisms regulating and orchestrating the function of these different cell types in the brain could make unique contributions to internal states and behavior that go well beyond the important contributions of neurocircuits. Accordingly, emerging molecular manipulation and imaging technologies, that together endow unprecedented cellular, spatial and temporal specificity, are being used to study the behavioral contributions of a number of molecular mechanisms in brain cell types other than neurons. The key thesis for a more molecularly oriented and broader systems neuroscience is that evolutionarily shaped molecular mechanisms in these cell systems may help explain and predict the vexing complexity of the brain’s internal states and behavior.

New technologies have traditionally been at the heart of the generation and evolution of new perspectives and transformative discoveries in science. Next, we will review an exciting and powerful new set of molecular imaging and manipulation techniques that are transforming multiple fields in biology, and that we propose are fueling the emergence of molecular systems neuroscience. These new techniques allow for the unprecedented temporal, cellular and subcellular manipulation and imaging of specific molecular mechanisms in targeted cell systems, circuits and brain regions of behaving animals.

New tools, methods and approaches unfailingly change a field by providing novel perspectives and opportunities for study. We identify three major areas of novel technology development that we predict will have a significant impact in systems neuroscience: (a) new developments in in vivo imaging techniques, including imaging of molecular mechanisms in specific cell types besides neurons, (b) new molecular sensors that can report on the activity of specific molecular mechanisms in a cell specific manner, and (c) new molecular actuators that can either activate or inhibit molecular mechanisms at a cellular or even sub-cellular level. These dramatic recent developments are opening systems neuroscience to more molecular and less neuron-centric perspectives.

### Imaging systems

From an imaging standpoint, one of the significant innovations of the last decade was the introduction of miniaturized head mounted fluorescent microscopes (miniscopes) that can record from hundreds of neurons (or other cells) for many days in freely moving and behaving animals, including mice [19–24]. This one-photon epifluorescence imaging approach uses a small CMOS (Complementary metal–oxide–semiconductor) sensor (similar to the technology in phone cameras), encased in a very light weight frame. The miniscopes also include an LED (light-emitting diode) that can be used for photo excitation of molecular sensors, such as calcium sensors.

Imaging with miniscopes involves removal of a small segment of the skull and possibly also brain tissue overlaying the imaging target, as well as the use of an implanted lens, such as a relay lens, to facilitate visualization of the target cells by the CMOS camera. Amongst other things, imaging with miniscopes facilitates the simultaneous visualization of a large population of cells, allowing for the detection of coordinated activity among groups of cells and thus identifying networks of communication within a cell population. Importantly, the nature of this type of imaging allows for longitudinal recordings of the same cells over several days, or even weeks [28, 29] in behaving animals. The size and weight of the miniscope allows for its use not just in mice and other rodents, but even in bats and birds. Constant improvements in miniscope design continue to reduce its size and weight and even allow for wireless recordings.

The ability of miniscopes to simultaneously assay at least 2 different emission/excitation spectra [26] enables not only imaging of two different molecular targets, but also simultaneous optogenetic manipulation and imaging by using one wavelength to excite the sensor’s fluorescence, and another wavelength to trigger an actuator (for example, inhibitory or excitatory opsins). This opened up the exciting possibility of tracking and manipulating molecular function in specific cells and circuits of behaving animals, such as mice. The combination of miniscope imaging and electrophysiological recordings with silicon probes, for example, allows researchers to simultaneously record very different properties of neurons and circuits during behavior. Information from electrophysiology and imaging complement each other well, due to the different strengths and weaknesses of these two approaches. For example, electrophysiology allows for recordings with much higher temporal resolution and for measurements of brain activity from large brain regions, while imaging enables stable longitudinal recordings of the same cells over many days. Studies comparing electrophysiological and calcium imaging data have already uncovered some unexpected findings. For instance, a recent report indicated that while calcium activity increased during sharp wave ripple trains, it actually decreased during sharp wave ripple singlets [30]. This shows that the combination of electrophysiology and imaging can yield experimental readouts that are data rich and complementary.

The relative ease of recordings, the inexpensive nature of miniscope systems, and consequently their widespread use in many laboratories world-wide, is fueling the engineering of new sensors with the required signal to noise ratios. Although miniscopes have been almost exclusively used for imaging changes in calcium concentration in targeted cells, such as neurons and astrocytes, new molecular sensor developments (see below) promise to expand this repertoire.

Recent advances in imaging go well beyond miniscopes and include other major advances which employ larger microscopes and head-fixed animals. The two-photon mesoscope is a powerful tool that allows an unprecedented number of neurons to be imaged at once [31]. The key feature of this technique is the combination of cellular resolution and a cylindrical field of view spanning several cubic millimeters in which cells can be imaged simultaneously in behaving head-fixed animals. Importantly, with this technique it is possible to simultaneously image from multiple brain areas. For example, a mesoscope was used to simultaneously image the somatosensory, parietal and retrosplenial cortices through a cranial window preparation. This technique is currently limited to imaging in head-fixed setups, which limits the array of behaviors that can be studied during imaging. However, the ability to record nearly simultaneously from a large number of neurons in different brain areas is a very important advance, and holds great promise for systems neuroscience since it considerably facilitates the longitudinal study of the interaction between multiple circuits in different brain areas, something that could not be easily accomplished with other techniques.

Holographic two-photon imaging can combine simultaneous imaging and selective optogenetic manipulation by taking advantage of multiple wavelengths. Importantly, this technique can specifically target voxels of the imaging field in a three-dimensional, volumetric manner to precisely manipulate and image individually identified neurons [32, 33]. The important implication for systems neuroscience is the possibility of targeting a subset of identified neurons with a specific activation profile within an imaged network, and determine the functional implications of these manipulations. For example, this approach was recently used to study how neurons are recruited into ensembles within the visual cortex, and how manipulation of these neurons within the ensemble affected the network as a whole, as well as performance in a behavioral task [25]. Surprisingly, the authors found that the manipulation of as little as 2 neurons could have a functional impact on network firing patterns and consequently behavior output! It is important to note that these ground-breaking approaches will undoubtedly be applied to molecular systems neuroscience studies since they could potentially be used to track and manipulate specific molecular systems (see below) in targeted circuits with very fine spatial precision. Novel imaging systems have been and will continue to be a key tool to investigate systems level neuronal dynamics in vivo.

### Molecular sensors and reporters

A recent revolution in molecular sensors and reporters has transformed the field of molecular imaging, and these powerful tools may very well be one of the foundation stones of molecular systems neuroscience. Fluorescence, autofluorescence and bioluminescence have been used with a plethora of different kinds of molecular reporters and sensors to explore molecular structure and function in an unprecedented manner. This revolution in molecular imaging is based on two complementary strategies: imaging of molecular structures and events by tagging them with photon emitting reporters, and the visualization of molecular mechanisms (e.g. receptor activation) by taking advantage of fusions with specific molecular sensors.

To visualize specific molecules, reporters such as fluorescent or bioluminescent proteins (e.g., Green Fluorescent Protein or GFP) are added so that they behave like “GPS devices” that track the movement of specific molecules in cells and circuits. Importantly, these tags are inert, and have a minimal impact on the endogenous properties of the targeted molecules, such as their activity, interaction with other molecules and movement in and out of cells. To precisely detect the subcellular distribution of a specific molecule by using just fluorescent tags like GFP, localization specific fluorescent tags have been developed. For example, the pH-sensitive GFP pHluorin can switch between a non-fluorescent state (quenched state) and a fluorescent state in response to changes in pH [34]. Fusing pHluorin to specific molecules makes it possible to track the movement of these molecules across subcellular compartments. For example, by fusing pHluorin to the α-amino-3-hydroxy-5-methyl-4-isoxazolepropionic acid receptor (AMPAR, one of the core transmitter receptors for synaptic transmission and plasticity) subunit GluA1 (thus generating SEP-GluA1) it was possible to generate a AMPAR that only becomes brightly fluorescent when this receptor moves to the relatively more alkaline environment of the postsynaptic membrane [35]. With this tool, it is now possible to easily track synaptic AMPAR trafficking after learning in vivo [36]. Another remarkable example is Synapto-pHluorin, a fusion protein that includes the transmembrane synaptic vesicle protein VAMP2 (Vesicle Associated Membrane Protein 2) and a variant of pHluorin called ecliptic pHluorin [37, 38]. At pHs characteristic of the milieu inside transmitter vesicles, synapto-pHluorin is non-fluorescent. When vesicles are released, synapto-pHluorin is exposed to the neutral extracellular space and brightly fluoresces, thus marking neurotransmitter release events. A modified version of a pH sensitive fluorine was used to study synaptic vesicle resting pool release and cycling during development [38] and the role of SNARE (Soluble N-Ethylmaleimide-Sensitive Factor Attachment Protein Receptor) proteins in regulating the dynamics of vesicle pool partitioning [39].

Since most GFP tagged proteins are continuously expressed, temporal information about the reported molecular events can be uncertain. To enable labeling within a specific time window, photoactivatable fluorescent proteins (PA-FPs) were developed [40, 41]. These fluorescent proteins display unique changes in their spectral properties upon exposure to a specific wavelength of light, thus providing a temporal landmark for the tracked proteins. Some PA-FPs can be activated from low fluorescent states to high fluorescence states [42, 43] while some can change from one fluorescent color to another [44, 45]. Others can be switched on and off reversibly [46, 47]. By applying the PA-FP kikGR (Kikume Green–Red), a unique imaging system was developed that combines Ca^2+^ imaging and engram identification to track engram activity [48]. Fuelled by a need to visualize molecular mechanisms in conjunction with network properties or behavioral output, two general categories of approaches with distinct temporal properties have been developed: (1) Sensors developed for live imaging; (2) sensors which need time for labeling. These sensors have been designed to track many different types of molecular events, including protein–protein interactions, receptor/enzyme activation, protein cleavage, gene transcription, etc. The design of these sensors has taken advantage of unique properties that define the targeted molecular events, including molecular conformational changes, dimerization and aggregation.

**Fig. 1:**
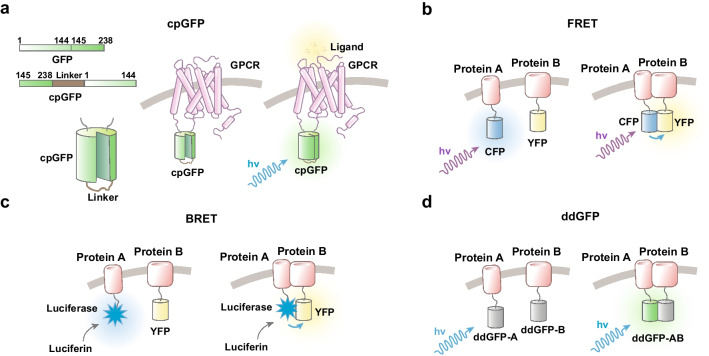
Different designs for real time sensors to detect molecular events. Here we use GPCR activation sensors as an example for cpGFP and protein–protein interaction sensors for the other sensors. **a** cpGFP: cpGFP is generated from GFP by genetically linking the original N- and C-termini with a short polypeptide linker, when the original protein is broken at specific positions (typically site 144). For GPCR sensor, cpGFP is inserted at specific locations between the transmembrane helix 5 (TM5) and 6 (TM6). Conformational changes by receptor activation cause alterations in the chromophore environment and change the fluorescence intensity. **b** FRET: A donor fluorophore (CFP) is fused to protein A and an acceptor fluorophore (YFP) is fused to protein B. Interaction between two proteins trigger energy transfer between CFP and YFP. Thus, violet excitation triggers yellow emission (YFP). **c** BRET: the donor fluorophore of the FRET technique is replaced by a luciferase, an enzyme which catalyzes luciferin oxidation to oxyluciferin, producing light emission and trigger YFP emission. **d** ddGFP: Weak or non-fluorescent ddGFP monomers (A and B) are separately fused to different proteins. Interaction between two proteins triggers the reversible association between monomers to form a fluorescent heterodimer

To observe molecular events in real time, sensors need to become brighter or change to a different fluorescent state immediately after such events occur (e.g. within milliseconds to seconds). To view G protein-coupled receptor (GPCR) activity, for example, a circularly permuted green fluorescent protein (cpGFP) was inserted at specific locations between the transmembrane helix 5 (TM5) and 6 (TM6) of the target GPCR (Fig. 1a); cpGFP is generated from GFP by genetically linking the original N- and C-termini with a short polypeptide linker (typically at site 144). The fluorescence of GFP remains unchanged but becomes more sensitive to the physicochemical environment [49–51]. The cp-GFP can be genetically inserted into a particular GPCR with the new N- and C-termini (TM5 and TM6) introduced close to the chromophore. Then, conformational changes driven by receptor activation cause alterations in the chromophore environment and a corresponding change in the fluorescence intensity, thus transducing information about GPCR activation into a fluorescent signal [52]. With this strategy, genetically-encoded GPCR-activation based (GRAB) sensors were developed for tracking in real time the activity of GPCRs, such as dopamine receptors [53], serotonin receptors and epinephrine receptors [54]. Based on a similar design, dLight1 was also developed to detect dopamine receptor activation [55].

Tools based on resonance energy transfer (RET) have often been used to visualize protein–protein interactions. RET is the principal mechanism for intermolecular or intramolecular redistribution of electron energy following molecular excitation [56]. Briefly, when two proteins are physically close to one another (within 1–10 nm), one protein (donor) can undergo a nonradiative (dipole–dipole) transfer of energy to the other protein (acceptor). In FRET (fluorescence resonance energy transfer), both proteins are fluorescent, whereas in bioluminescent resonance energy transfer (BRET) the energy transfer is typically between a luciferase and a light-sensitive molecule (usually a fluorescent protein, Fig. 1b and c). When the two proteins are close enough, excitation of the donors, either by light or by a substrate, will trigger emission events from the acceptor domains/proteins. Several sensors have been made based on RET. For example, both BRET and FRET have been used to describe the molecular dynamics of Tyrosine receptor kinase activation such as TrkB, the Tropomyosin receptor kinase B [57, 58]. To precisely determine the interaction between neuronal activity patterns and transcription factor activity, a FRET based CREB (the cAMP-response element binding protein) sensor was used together with GCaMP6s in the visual cortex to study how experience shapes the interplay between CREB and neuronal activity in the neocortex of awake mice [59].

Despite the remarkable achievements made possible by FRET technology, FRET sensors have encountered several challenges in in vivo imaging. These include the low sensitivity and efficiency of FRET sensors which limit in vivo applications, the need for two different fluorescences for donor and acceptor for a single signaling event, thus limiting the number of events detected. By utilizing dimerization-dependent fluorescent proteins (ddFPs, Fig. 1d), multi-colored and intensiometric biosensors were developed to visualize the activity of multiple small GTPases [60]. Using two-photon imaging coupled with blue light-based optogenetic modules to activate FGFR1 and TrkB signaling (OptoFGFR1 and OptoTrkB, respectively, see below), these studies showed that red fluorescent sensors can be used to visualize local and reversible changes in the activity of small GTPases both in cultured neurons and in the mouse brain.

**Fig. 2:**
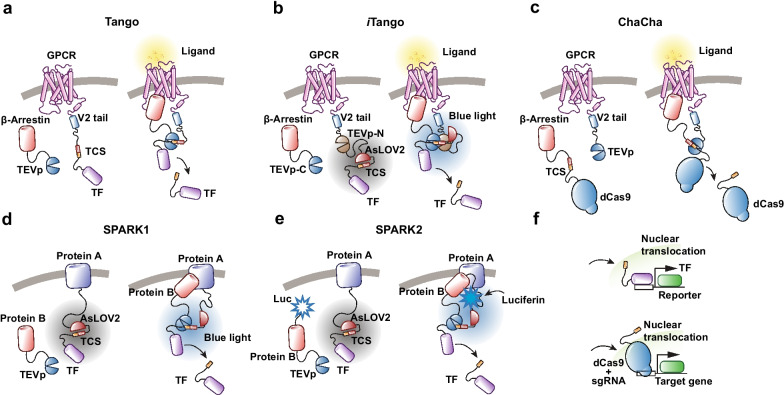
TEV protease-based sensor design. Different designs for TEV protease-based sensors to detect molecular events. GPCR activation sensors are used as examples for Tango, iTango and ChaCha system. Protein–protein interaction sensors are used for SPARK1/2 system. **a** Tango system: TCS and a transcription factor (TF) are fused to the c-terminal of a GPCR. Upon activation, β-Arrestin tagged with TEVp will bind intracellular loop of GPCR and cleave TCS when exposed to release TF. **b** *i*Tango system: TF is coupled to the C-terminal of a GPCR via TCS, which is caged by AsLOV2/Jα. Upon activation, β-Arrestin tagged with TEVp-C will bind intracellular loop of GPCR tagged with TEVp-N, which will form functional TEV and cleave TCS when exposed to light stimulation and release TF. **c** ChaCha system: The TCS and a dCas9 based TF are fused to β-Arrestin while TEVp is fused to the C-terminal of a GPCR. After interaction of β-Arrestin and GPCR upon activation, TCS is cleaved and dCas9 is released. **d** SPARK1 system: Protein A is fused to the AsLOV2 caged TCS and TF. Protein B is fused to TEVp. Upon interaction between proteins A and B, blue light can dismiss the protection from LOV domain and leave TCS cleaved by TEVp, which then release TF. **e** SPARK2 system: Similar to SPARK1 design. Instead of using blue light, luciferase is fused to protein B. After interaction between proteins A and B, the luciferase with luciferin activates the LOV domain via BRET, allowing the TEVp to cleave TCS and release TF. **f** Released TF translocate into nuclear and initiate reporter gene expression. Notably, dCas9 with sgRNA can easily modify endogenous gene expression other than reporter gene

In addition to fast changes in photon emissions, some tools, such as the Tango system (Fig. 2a), track molecular events (e.g., GPCR activation) by initiating the expression of a reporter gene [61]. In the Tango system, a tetracycline-controlled transactivator (tTA) is coupled to the target GPCR and cleaved following ligand binding by recruitment of a TEV protease (Tobacco Etch Virus nuclear-inclusion-a-endopeptidase) fused to β-arrestin. The TEV-tTA is then transported to the nucleus where it triggers the transcription of a reporter gene (e.g., GFP) under the regulation of a TRE (tetracycline responsive element) promoter. A light sensitive AsLOV2 domain (LOV2 domain of Avena Sativa phototropin 1) that protects the TEV cleavage site (TCS), confers temporal control of Tango signaling through photomodulation [58] (Fig. 2b). The iTango systems has been used to track the activation of the dopamine and the oxytocin receptors in vivo [62, 63].

In a similar system, Chacha [60], the effector (e.g., tTA) is fused to the C-terminus of the β-Arrestin (with a TEV site), and the TEV protease is bound to the C-terminus of the GPCR. Activation of the GPCR leads to the proteolytic cleavage of the tTA effector bound to β-Arrestin, which is then free to activate the transcription of the reporter (e.g., GFP, Fig. 2c).

SPARK (Specific Protein Association tool giving transcriptional Readout with rapid Kinetics, Fig. 2d and e) is a related system in which proteolytic release of a membrane-tethered transcription factor requires both a protein–protein interaction to deliver a protease proximal to its cleavage peptide, and blue light to uncage the cleavage site. SPARK has been used to detect a wide range of different protein–protein interactions. SPARK2 [64] incorporates a luciferase moiety to modulate the light-sensitive LOV (Light-Oxygen Voltage) domain. Thus, SPARK2 can be temporally controlled by either light or by addition of luciferin (activates luciferase), thus causing luciferase to change the configuration of LOV, via proximity-dependent BRET, and promote proteolytic release of the transcription factor. This system reportedly shows lower-background noise and improved specificity compared to the systems described above.

The sensors and reporters described above could be used to study the orchestration of specific molecular events in cellular systems, such as neurocircuits. At the systems level, some of these sensors have already been used at scales that allow monitoring of entire brain regions in vivo when combined with endoscopic imaging techniques, or at lower resolution through fiber photometry. Among others, neuromodulatory activity has been studied at a region-wide scale [53–55], or brain glucose levels have been imaged in awake behaving animals [65].

Insights into these molecular systems events could be key in discerning how these systems modulate behavior. Since these methods use engineered genetic sensors to detect molecular events of interest, any discovery made with any one of these approaches would have to be confirmed with other discrete but complementary methods that for example look at undisturbed systems, such as immunostaining, in situ hybridization, etc. This would help to confirm that the findings obtained are not the result of the genetic engineering systems used, and actually reflect the biology of the phenomena being studied (e.g., attention, learning, memory, etc.). Importantly, there are a number of novel molecular approaches that can be used to manipulate specific molecular mechanisms in specific cells and systems, and at specific times. These approaches are especially important in testing hypotheses derived from the reporters and sensors just described (and vice-versa). As always, the combination of diverse manipulation, observation and modeling tools is critical to confirm and understand any new biological finding.

### Optogenetic manipulation of molecular systems

Historically, molecular manipulation tools have their roots in pharmacology (e.g., drugs) and genetic approaches (e.g., transgenics, knock outs, viral manipulations). Over time these approaches have become more and more specific, and therefore more easily applicable to systems neuroscience questions. For example, region specific drug infusions through cannulae and osmotic pumps allowed targeting of drugs to specific brains areas, while conditional knock outs, the use of cell-specific promoters and viral vectors dramatically enhanced the spatial and temporal specificity of genetic approaches. More recently, light-mediated modulation of specific molecular systems has revolutionized our ability to manipulate molecular pathways in specific cells of targeted circuits and at times of our choosing. These developments have had a key role in bringing molecular approaches to systems neuroscience. Optical manipulation tools, for example, have been developed to target almost every step of cellular signaling events, from ligand binding, receptor activation, associated molecular signaling, all the way to downstream gene expression, and protein translation. Importantly, these molecular manipulations tools are perfect complements to the molecular reporting tools described above. Convergent findings with manipulation and reporting tools are key for discoveries in molecular systems neuroscience. Below, we will review some of the key approaches in the fast-evolving field of molecular manipulations tools which we propose will have an incalculable impact in systems neuroscience.

### Targeting receptor activity with light

Receptor activation is the first step of many molecular signaling pathways. Receptors play essential roles in translating extracellular signals into intracellular signaling events, and therefore they provide a key site to manipulate cell systems. Natural ligands, as well as agonists and antagonists, have been traditionally used to specifically modulate receptor activity and study related mechanisms. Unfortunately, these classic pharmacological tools cannot be selectively targeted to specific cells in a circuit, and their temporal resolution is limited by the time required for drug diffusion and degradation, making them difficult or impossible to use in system neuroscience studies. Below, we will review ground-breaking new molecular tools that circumvent these limitations, and therefore bring molecular manipulations to the purview of systems neuroscience.

Optical modulation combined with sophisticated genetic approaches have made it possible to regulate receptor function in specific cells of targeted systems and in defined brain regions. Receptors for neurotransmitters, such as glutamate, GABA (Gamma-aminobutyric acid), dopamine, serotonin and noradrenaline have played a central role in neuroscience. Thus, the novel optical tools modulating the activation of these receptors provide ground-breaking opportunities to explore hidden properties of related systems with spatially and temporally precise receptor manipulations.

#### Targeting ligands

Since most receptors need their ligands for activation, temporal and spatial regulation of ligands has been a key strategy to regulate receptors. Optopharmacological approaches include a number of photosensitive reagents that act on a wide range of molecules, including channels and receptors [66].

Caged ligands, whose release is controlled by light, were a key developing milestone in the field. Caged molecules have photolabile protective moieties that cage the molecule in question, and keep it from interacting with its partners (e.g., a receptor). Light activation removes the molecular cage, enabling the uncaged ligand to bind to its receptor. When caged, the compound/ligand stays inert until illuminated by the activating light wavelength. Uncaging following illumination, can trigger a rapid increase in the concentration of a specific ligand, therefore resulting in the prompt activation of a given receptor. Since uncaging is controlled by light, it can be restricted to specific sites and cells of targeted circuits. Furthermore, with two-photon light sources, uncaging can be directed to very specific sub-cellular sites, such as dendritic spines, thus significantly enhancing the spatial and temporal specificity of molecular manipulations [67]. Currently, there are caged ligands for most neurotransmitters, including glutamate [68, 69], GABA [70], dopamine [71] and serotonin [72], to name a few. Furthermore, caged agonists and antagonists of neuropeptide receptors are also available [73–75], thus allowing for light control of key neurotransmission and neuromodulation events in neurocircuits.

Photoswitchable ligands are another key class of light controlled ligands. These ligands allow for bidirectional regulation (activation/deactivation) with precise time control. Unlike caged compounds, photoswitchable ligands can reversibly alternate between an active and an inactive state in response to two different wavelengths of light [67], and have been designed for a large variety of neurotransmitter receptors and ion channels, including metabotropic glutamate receptors [76–78], ionotropic glutamate receptors [79, 80], GABA receptors [81, 82], K^+^ channels [83, 84] and Ca^2+^ channels [85].

Uncaging as well as photoactivated ligands have been widely used for more than a decade to study synaptic transmission, compartmentalization of vesicles and receptors at the synapse, as well as synaptic plasticity [86]. It is also worth mentioning that photopharmacology has, for example, been used to restore light-sensitivity in amacrine and retinal ganglion cells, as well as to develop alternative approaches to vision restoration [80, 87]. These studies include in vivo monitoring of local changes in synaptic morphology and synaptic pruning which can then be used to infer mechanisms underlying the activity and connectivity of the network at a systems level [68, 69, 88, 89].

To avoid off-target effects, it is critical that caged and photoswitchable ligands are inert before activation, since high concentrations are always needed to ensure sufficient levels of these ligands around the targeted receptors. To address this challenge, photoswitchable ligands have been covalently tethered to receptor binding pockets, which result in very high concentrations near the sites of intended action (i.e., near receptors), but comparatively lower concentrations elsewhere [67]. Photoswitchable tethered ligands have been developed for a variety of receptors, including the ionotropic glutamate receptor, iGluR [90, 91], the metabotropic glutamate receptor, mGluR [92, 93], GABAa receptor [94, 95], the nicotinic acetylcholine receptor, nAChR [96] and Dopamine receptor [97].

#### Reengineering receptors

Efforts to control the function of receptors in real time have also taken advantage of hybrid receptors that incorporate, for example, the extracellular domains of opsins (so that the receptors become light activated) and that preserve the effector intracellular domains of the receptor. For example, since GPCRs share conserved structure, the extracellular loops of several GPCRs have been replaced with those of rhodopsin so that, upon light stimulation, the structure of the transmembrane domains of rhodopsin changes, thus activating downstream G protein complexes normally associated with the targeted GPCR [98]. This approach is termed Opto-XR [98, 99].Based on this design, a number of optoXRs have been engineered, allowing for optogenetic control of adrenergic receptors [100], adenosine receptors [101], mGluRs [102], dopamine receptors [103], and serotonin receptors [104]. Although Opto-XR engineering has so far been restricted to GPCRs, this approach could potentially be applied to other receptors.

**Fig. 3:**
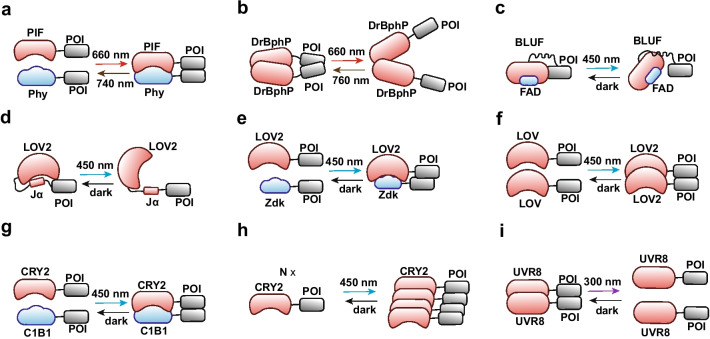
Commonly used photoactivatable systems. Brief scheme of properties and conformational changes of several photoactivatable systems. Here we use POI (protein of interest) to represent the effectors fused to the photoactivatable protein. **a** Phy-PIF system: Exposure to FR (640–680 nm) light induces association of PIF and Phy, while exposure to NIR (740–780 nm) light induces dissociation of PIF from Phy. **b** DrBphP system: Under NIR (740–780 nm) light, DrBphP can form homodimers. After Absorption of FR (640–680 nm) light, DrBphP dimers come apart. **c** BLUF system: Blue light (~ 450 nm) can trigger conformational change of BLUF and expose/activate POI. **d** LOV2/Jα system: Interaction between Light-sensitive LOV2 domain of and Jα helix can be reversibly disrupted by blue light (~ 450 nm). **e** LOV2TRAP system: Zdk binds selectively to the dark state of LOV2, which can be separated apart by blue light (~ 450 nm). **f** LOV2-LOV2 system: LOV2 form homo-dimerization upon blue light stimulation (~ 450 nm). **g** CRY2-CIB1 system: Exposure to blue (~ 450 nm) light induces association of CRY2 and CIB1. **h** CRY2 oligomerization: In the presence of blue light (~ 450 nm), photoexcited CRY2 undergoes photoreduction to adopt an open conformation, leading to the formation of CRY2 homodimers and homo-oligomers. **i** LOV2 form homo-dimerization at dark state, which come apart upon UV light (~ 300 nm)

Ion Channels and other proteins can also be regulated by linking them to a class of proteins that changes conformation in response to light (Fig. 3). These photoactivatable proteins include LOV, Cryptochrome (CRYs), Blue-light-using flavin adenine dinucleotide (BLUF), Phytochrome (Phy), and UV-B (Ultraviolet-B radiation) photoreceptor UVR8 (UV Resistance Locus 8). The conformational changes induced by blue light in LOV2, for example, were used to switch a K^+^-channel (BLINK1, blue-light-induced K^+^ channel 1) between an open or closed state [105]. LOV 2 and CRY2 were also used to control dimerization/or oligomerization, and therefore the activation, of receptor tyrosine kinases (optoFGFR1, light activated fibroblast growth factor receptor 1; [106–108]). Similarly, a photoactivable domain, inspired by Arabidopsis thaliana cryptochrome 2, was engineered to allow blue-light activation of Trk signaling in a cell-specific and spatially directed manner [109, 110]. OptoTrkB was also used to activate receptor signaling in the mouse brain [111]. An Optogenetically activatable Fas receptor (optoFAS) was developed using the blue light-induced homo-oligomerizing property of cryptochrome 2 (CRY2). Activation of this receptor in immature neurons of the dentate gyrus revealed a role for Fas signaling in the elevation of mTOR and ERK levels in these neurons as well as the subsequent induction of adult hippocampal neurogenesis [112].

CLICR (Clustering Indirectly using Cryptochrome 2), which again uses engineered Arabidopsis cryptochrome 2, is a modular approach designed to allow optic control of the clustering of a range of transmembrane receptors [113]. This is important because clustering has a key role in the activation of many receptors. CLICR was used to allow optical control of integrins, platelet-derived growth factor and fibroblast growth factor receptor [113].

Although many of the methods mentioned in this section have already been used in neuroscience experiments, they all have specific individual disadvantages that somewhat limit their application. For example, caged ligands, as well as photoswitchable ligands, require that the ligand be exogenously supplied. Unless the ligand can be expressed by cDNA viral vectors at the desired levels, the high dosages required for these experiments can sometimes result in off-target effects [66] which complicate in vivo applications. In this respect, it is important to note that tethered photoswitchable ligands successfully avoid this problem by dramatically reducing the probability of off-target effects. Thus, tethered photoswitchable ligands hold great promise for in vivo studies, including molecular system neuroscience experiments.

Similarly, although the OptoXR approach can be widely used to tightly manipulate GPCR function, their highly conserved structures and signaling mechanisms do not always represent the diversity of extracellular and transmembrane domains seen in endogenous GPCRs. For example, some GPCRs form homodimers or heterodimers, and this can significantly change their downstream signaling, thereby affecting the precision and usefulness of the OptoXR approach [114–116]. Similarly, interactions with other receptors (e.g. Receptor Tyrosine Kinases) are also critical components of GPCR signaling, and this is not captured by the OptoXR system [116, 117]. As a result, this approach only mimics one aspect of GPCR activation (i.e., G protein signaling), leaving out other critical aspects of the activation of these receptors. Furthermore, all approaches that require the cellular expression of specific molecules always face the possibility that the overexpressed molecule unintentionally interferes with one or more functions of the targeted molecule. For example, GPCRs have constitutive (also known as intrinsic or basal) levels of activity in the absence of their ligands. Consequently, their overexpression may abnormally amplify downstream signaling and thus affect OptoXR-base manipulations. OptoXR technology has been used to study the activity of several brain regions and systems. For example, OptoXR has been used with a modified type 1 dopamine receptor in the nucleus accumbens (NAc) to test its role in social behavior [103]; It has also been used for a photoswitchable GPCR based opsin to inhibit neurotransmitter release at NAc axon terminals and suppress reward-seeking behavior [118]. OptoXRs are also a convenient tool to study the interactions between a brain region and a neuromodulatory input, as they allow for the silencing of the neuromodulatory input and simultaneous re-establishment of the neuromodulatory downstream pathway specifically in the targeted region through optogenetic activation of the OptoXR.

### Targeting intracellular mechanisms

In addition to receptor function, there are several signaling molecular mechanisms that can be manipulated for the study of cells and systems. These include protein interaction, phosphorylation, cleavage, aggregation/disaggregation, allosteric changes, etc. A number of different optogenetic tools have been designed to manipulate specific aspects of these signaling mechanisms. Next, we will review some examples that illustrate the enormous creative potential of these approaches.

#### Optogenetic control of molecular localization and association

Protein–protein interactions (PPI) are critical for intracellular signaling. These interactions are required to form complexes that perform particular functions (e.g., Cdk5, Cyclin Dependent Kinase 5 and P35/P25 complex [119]). For example, certain enzymes (e.g. kinases) and downstream effectors (e.g. rapidly accelerated fibrosarcoma kinase or RAF and Erk) are recruited for signal amplification [109]; while other complexes target proteins to subcellular regions (e.g. A-kinase activity reporter, AKAR targets Protein kinase A) thus spatially restricting intracellular signaling [110]. Accordingly, tools for optogenetic induction of molecular dimerization, aggregation or disaggregation could be used to modulate as well as modify protein interactions and consequently the properties of cells and systems.

To control protein–protein interactions optogenetically, several Light-Inducible Dimerization (LIDs) systems have been developed that take advantage of light-sensitive domains including LOV2, Cryptochrome 2 (CRY2)- Cryptochrome-interacting basic-helix-loop-helix (CIB1), Phytochrome (Phy)- Phytochrome-interacting factor (PIF) and Deinococcus radiodurans bacterial phytochrome (DrBphP, Fig. 3, Table 1) [120–128]. These light sensitive domains change conformation after light activation, and in the right molecular configuration, this property can be used to modulate specific protein–protein interactions. For example, a Light-Inducible Dimerization system based on cryptochrome (CRY2)–cryptochrome-interacting basic-helix-loop-helix (CIB1) was developed to optogenetically control the activation of Raf/Erk and PIP3 (Phosphatidylinositol (3,4,5)-trisphosphate) signaling [129, 130]. LARIAT (light-activated reversible inhibition by assembled trap) and IM-LARIAT (light-activated reversible inhibition by assembled trap of intracellular membranes) are sophisticated systems that take advantage of CRY2- CIB1 interactions. In this case, light activation triggers fast and reversible protein–protein aggregation. This system has been engineered to control a number of signaling systems, including Rab-mediated intracellular membrane trafficking, a phenomenon involved in critical cellular processes [131, 132]. Ras/ERK and Rho GTPase signaling can also be optogenetically modulated by Light-inducible dimerization of Phytochrome B (PhyB) and phytochrome-interacting factor 6 (PIF6) domains [133]. Tunable Light-Inducible dimerization tags (TULIPs) and LOV2 Trap and Release of Protein (LOVTRAP) have also been developed to optogenetically control protein translocation using the LOV2 system [134].

**Table 1:** Summary of commonly used light inducible systems

Light-inducible systems	Description	Applications
CRY2-CIB	CRY2 undergoes blue light–dependent interaction with CIB1, which mediates light responses in plants [123]	1) Optogenetic control of PIP3 [130] 2) Light-induced activation of the Raf/MEK/ERK [129] 3) LARIAT [132] and IM-LARIAT [131] 4) Opto-TrK [60, 109]
CRY2-CRY2	CRY2 undergoes homo-oligomerization upon blue light stimulation [183]	1) OptoFGFR1 [107] 2) OptoFAS [112] 3) CLICR [113]
Phy-PIF	Exposure to 650 nm induces association of PIF and Phy, while exposure to 750 nm light induces dissociation of PIF from Phy [184]	1) Opto-SOS [133, 185] 2) Spatiotemporal control of the Rho GTPase signaling [150]
LOV2	Interaction between Light-sensitive LOV2 domain of a Jα helix can be reversibly disrupted by blue light [124]	1) BLINK1 [105] 2) LOVTRAP [134] 3) TULIPs [186] 4) Light induced nuclear translocation [154–158]
LOV2-LOV2	LOV2 undergoes homo-dimerization upon blue light stimulation [187]	1) EL222 [126, 188] 2) OptoFGFR1 [108]
DrBphP	DrBphP is from Deinococcus radiodurans bacterial phytochrome, the dimeric photoreceptor proteins that sense red light levels. Under NIR (740–780 nm) light, DrBphP can form homodimers. After Absorption of FR (640–680 nm) light, DrBphP dimers come apart [125]	1) Opto-RTK [127, 128] 2) Light-Activated Cyclic-Mononucleotide Phosphodiesterases [189]

Here, we listed several commonly used light inducible system as well as examples of how these systems were used in the regulation of molecular events

Some of these tools were already applied to neuroscience research. Studies using CRY2-CIB1 based OptoRAF1 in neuronal progenitor cells showed that optogenetic RAF activation promotes astrocytogenesis in mouse neural progenitors [135]. Light-inducible dimerization was used, among other things, to study the trafficking mechanisms of GPCRs and their regulatory proteins, and in particular β-arrestin mediated trafficking [136, 137]. In another example, IM-LARIAT was used to reversibly disrupt membrane aggregation of Rab-GTPases to study differences in the trafficking properties mediated by different Rab-GTPases in several cell types, including hippocampal neurons [131]. There are countless systems level experiments that could be carried out with these approaches. For example, it would be interesting to combine two-photon all optical approaches with light-inducible PPI to image the changes of neuronal networks within a region during disruption of physiological localization and trafficking of key synaptic proteins. Such an experiment could highlight the impact of synaptic protein trafficking to the patterns of acitivity of a specific neuronal assembly during learning.

### Optogenetic control of endogenous Ca^2+^ channels in vivo

Intracellular Ca^2+^ signaling in neuronal cells plays a fundamental role in translating diverse extracellular inputs into complex brain functions, such as learning, memory formation and emotion. Previously, the Heo group developed a blue-light–responsive Ca^2+^ channel modulator, OptoSTIM1, that triggers the opening of Ca^2+^-release–activated Ca^2+^ (CRAC) channels, and successfully demonstrated for the first time that inducing Ca^2+^-selective signaling in excitatory neurons of the hippocampal CA1 region is capable of enhancing contextual fear memory in mice [138]. They also recently developed the next generation of optogenetic Ca^2+^ modulators, termed monSTIM1 (monster OptoSTIM1), that show ultra-sensitivity to blue light [139]. Using structural predictions of the dimeric interface of cryptochrome 2 (CRY2), they identified a single point mutant (CRY2^E281A^) that exhibits stable basal intracellular Ca^2+^ concentrations (regardless of protein expression levels) and drastically enhanced photosensitivity when coupled with a superior CRY2 clustering system (CRY2clust). With MonSTIM1 it was possible to induce Ca^2+^ signaling in excitatory neurons or astrocytes in the cortex, hippocampus and thalamus of awake mice with non-invasive light simulation. When applied to the anterior cingulate cortex (ACC) of the mouse brain, monSTIM1 effectively enhanced short-term (4 min) and long-term (24 h) memory for observational fear in mice. This experiment directly demonstrated how the fine tuning of Ca^2+^ signaling in a single brain region can effectively affect memory encoding.

#### Light modulation of enzyme activity

In addition to controlling dimerization/aggregation, optogenetic strategies have also been focused on controlling enzyme activity by inducing light-triggered changes in protein conformation that result in activity changes in targeted proteins. For example, light induced structural changes in AsLOV2 were used to change the activity of Ras-related C3 botulinum toxin substrate 1, Rac1 (PA-Rac1), a small signaling GTPase involved in a diverse array of cellular functions. Briefly, the complete LOV2-Jα sequence was fused to the amino terminus of a constitutively active Rac1. Before light activation, the LOV domain tightly binds the Jα helix domain, and this prevents Rac1 effectors from binding to Rac1. Light stimulation unwinds the Jα helix, which releases steric inhibition of Rac1, allowing this protein to bind to its effectors [131]. Amongst other things, this tool could be used for rescue experiments to determine the molecular pathways through which one circuit maight affect plasticity in a downstream circuit.

#### Optogenetic control of gene transcription and translation

Other than controlling intracellular signaling, opto-tools have also been engineered to modulate gene expression. Some of these tools take advantage of transcription systems such as Cre/Flippase (Flp) recombinase or tetracycline system, that have been used to control the expression of target genes under the control of the artificial promoters of these systems, while others were designed to directly bind targeted promoters and thus modulate the expression of the associated genes. Site specific recombinase systems, such as Cre/locus of X-over P1 (Loxp), Flp/Flippase recognition target (FRT), are widely used for cell-type specific gene modulation by expressing these systems in transgenic mice or by delivering them with viral vectors [140]. Although these approaches are widely used, and some versions are even pharmacologically inducible by agents such as tamoxifen or 4-hydroxytamoxifen, they lack the temporal precision [141] required for some system neuroscience purposes. To improve on temporal precision, photoinducible Cre, Flp (PA-FLP) [142–145] and tet systems were developed [146, 147], that allow for tight regulation by light. Beyond temporal control, these systems also remediate problems with diffusion, and off-target effects due to the administration of a pharmacological inducer. Importantly, damage caused by optical cannula implantation can also be avoided by using approaches such as near infrared deep brain stimulation and ultrasensitive opsins [142, 148, 149].

Nuclease-dead Cas molecules (dCas9 and dCas12a), that still bind DNA guided by single guide RNA (sgRNA), have been fused to light induced dimerizers (e.g., CRY2-CIB1, PhyB / PIF and Magnet pMag-nMag), such that light stimulation triggers the expression of targeted genes. RNA based targeting makes this system exquisitely specific. Similar strategies, in which light induced dimerizers were fused to specific transcription factors, have also been used to optogenetically control the transcription of target genes [150–153]. Since transcription regulates a plethora of cell and circuit states, such as memory stabilization, these optogenetic tools could be used to study a number of system properties.

Another strategy to optogenetically control gene transcription takes advantage of cellular mechanisms that guide transcription factors to the cell nucleus where they modulate gene expression. Briefly, these mechanisms involve small protein segments that function as cell nuclear export signals (NES) and nuclear localization signals (NLS) to control the cellular localization of specific transcription factors [154]. For example, light induced conformation changes, mediated by using LOV and CRY2, expose NLSs and hide NESs so that the engineered transcription factor can enter the cell nucleus and initiate gene expression [134, 155–158].

Subcellular localization of RNA also plays a key role in the function of neurons, such as the targeting and local translation of RNA at synapses. A CRISPR(clustered regularly interspaced short palindromic repeats)/Cas9 derived system (RNA-targeting Cas9 or RCas9) has also been used not only to visualize the trafficking of specific mRNAs in cells (with EGFP fusions), but also to optogenetically modulate the trafficking of specific RNAs by trapping (or releasing) these mRNAs in molecular clusters within the cell [159]. Functionally, this optogenetically regulated sequestering approach reduces the ability of target mRNAs to get access to the cell’s translation machinery (i.e., ribosomes), thus markedly attenuating the synthesis of specific proteins [159]. Remarkably, this mRNA sequestering strategy can be targeted to different cellular compartments so that, for example, synaptic translation of a specific gene is disrupted, but its somatic translation is not. Optogenetic control of gene transcription has been used to study signaling pathways associated with calcium stress, by artificially manipulating the levels of nuclear translocation of the transcription factor Calcineurin-responsive zinc finger 1 (Crz1). The temporal resolution of this optogenetic technique was key in these studies [160]. This powerful new tool promises to be very useful in molecular systems neuroscience studies. Not only does it allow for the precise temporal control of protein translation through tightly regulated optogenetic release of RNA, it also affords exquisite spatial control since it can be used to specifically target mRNA sequestering in different subcellur comparments. For example, this tool could be used to examine the role of somatic versus synaptic translation of proteins at specific times during learning or memory consolidation, and in different cellular structures and cell types in the brain.

### Modulation of protein function by optogenetically activated nanobodies

The ability of monoclonal antibodies to very specifically target their antigens has been widely used in research and in medical treatments (e.g. cancer therapy). However, their large size (150 kDa), and non-covalently associated variable domains, make it essentially impractical to express such large proteins with cDNA constructs, limiting their usefulness for many applications, including systems neuroscience. In contrast, nanobodies (natural single domain antibodies*)*, proteins ten times smaller (~ 15 kDa) than an antibody, have comparable affinities for their antigens but are highly soluble, stable, and have excellent tissue penetration, thus making them useful as modulation tools for protein function [161, 162]. Importantly, the light activated properties of AsLOV2 were combined with nanobodies (opto-nanobodies), such that light stimulation triggers a conformational change that alters the opto-nanobody’s antigen binding (either increases or decreases), thus allowing control over the function of the targeted protein [163]. Additionally, magnet optical dimerization tools (nMagHigh1 and pMagHigh1) were used to engineer a light activated nanobody system (optobodies) that can be expressed in target cells as intracellular antibodies (intrabodies), which can bind and inhibit their targets after light stimulation (e.g. β2 adrenergic receptor optobodies) [164]. Furthermore, a chemogenetic control of nanobodies was also developed (ligand-modulated antibody fragments or LAMAs and chemobodies based on rapamycin-induced FRB-FKBP dimerization of split nanobody fragments) [164, 165].

### Molecular inactivation with light triggered free radicals

Reactive oxygen species (ROS) can be used to inactivate DNA, RNA, lipids and proteins. With Chromophore-Assisted Laser or light Inactivation (CALI), it is possible to optogenetically produce ROS and inactivate a number of intracellular molecules in cell sites targeted by light. Because the free radical species generated during CALI are short-lived (less than 1 s), and light can be limited to small subcellular regions, this approach is an important spatially and temporally controlled loss-of-function tool [166, 167]. Although CALI initially used dyes for free radical production, fluorescent proteins such as EGFP [168], and more recently KillerRed, have been combined with CALI to specifically optogenetically inactivate proteins fused to either of these two photosensitizers [169, 170]. These approaches can then be used to test the effects of transient inactivation of a single protein at the systems and behavioral level. For example, CALI was used in the hippocampus to transiently inactivate actin polymerization, disrupt LTP and impair context-specific memory. Subsequently, miniscope imaging and CALI based inactivation techniques were combined to show distinct roles of hippocampal online and offline LTP in engram formation [171].

### What is next?

The development of opto tools has been centered on two key aspects: (1) better spatial and temporal specify and (2) novel combinations of molecular systems, which continue to open novel modulation opportunities.

Although optogenetics enable the modification of neuronal activity with high spatial and temporal resolution, there are limitations that need to be addressed. For example, light delivery often involves the implantation of optic fibers, and this inevitably results in some damage to the targeted tissue, especially when deep brain regions are studied (e.g. hypothalamus), or when multiple-areas need to be modulated. Deep tissue penetration by light is another challenge that needs to be addressed. Opsins with red-shifted spectra have been developed to allow possible light delivery through the skull and to deeper brain areas [33, 172]. Another solution is to combine traditional opsins with upconversion nanoparticles (UCNPs). These nanoparticles can be transcranially stimulated by near-infrared light and, as a result, they emit light in the visible spectrum required to excite most opsins [149]. However, high power light sources still need to be used to ensure sufficient activation of the opsins involved. This can result in unwanted increases in the temperature of local tissues, and consequently inflammation and even cell death. Strong illumination can alter the function of specific neurons independently of opsin expression, making the further development of luciferase-based bioluminescence systems a very promising alternative.

Bioluminescence involves the emission of light as a result of a cellular biochemical reaction. In contrast to fluorescence and phosphorescence, bioluminescence reactions do not require exogenous light activation. Instead, in bioluminescent systems the excitation light is produced through the oxygenation of a substrate, generically called luciferin, and the activity of the enzyme luciferase [173–175]: a luciferin injection, together with appropriately expressed luciferase, can trigger the light emission required for opsin activation. Since in this case the light source (i.e. the luciferin/luciferase combination) is designed to be extremely close to the opsin, very low power is needed to trigger optogenetic activation, thus avoiding damage associated with heating that can take place in traditional optogenetic systems. For example, luciferase has been fused to channelrhodopsin to excite neurons (luminescent opsin, LMO) or to a proton pump to inhibit these cells (inhibitory LMO, iLMO) [176]. Unlike traditional chemogenetic approaches, where drugs such as CNO/clozapine are known to have significant side effects, luciferin seems to be relatively inert in cells [177, 178].

Although bioluminescent approaches suffer from inferior temporal resolution (compared with classical optogenetic methods), the spatial resolution of these systems can be superior because illumination comes from light sources with cellular and even molecular resolution. Luciferase can be expressed in any target cellular/subcellular region, and only the nearby opsins receive enough photons to be activated. Therefore, bioluminescent derived systems allow the investigation of questions that require optogenetic modulation at higher spatial resolutions. For example, luciferase has been combined with the SPARK2 system, which can label beta2 adrenergic receptor activation, so that only cells closely interacting with the luciferase expressing cells are activated [64].

Another limitation of current optogenetic systems is that the enhancement and inhibition of molecular/cellular activity is done arbitrarily at a time chosen by the experimenter, and not as a result of some molecular, cellular or circuit event intrinsic to the systems being studied. To circumvent this limitation, molecular sensors have been combined with optogenetic modulators so that specific biological phenomena perceived by the sensor triggers when optogenetic activation takes place. For example, naturally occurring neural dynamics during behavior can provide the trigger for subsequent precisely timed optogenetic manipulation to mimic or block endogenous activity. GCaMP6 based fibre photometry was used to identify differences in NAc D2 dopamine receptor-expressing neuronal activity during the decision process. Optogenetic stimulation was applied only during the decision period to mimic endogenous dynamics of single-trial control of risk-seeking behavior [179, 180].

As an extension of this idea, the promoter of the activity dependent immediate early gene c-Fos has been engineered to control the transcription of a light controlled tTA (opto-tTA) so that the transcription of specific genes is both activity and light-dependent. Importantly, unlike conventional tTAs [181], the regulation of opto-tTA does not involve doxycycline. Briefly, neuronal activity turns on the cFos promoter, which in turn will guide the initiation of the expression of opto-tTA. After optogenetic activation, opto-tTA will bind and activate TRE, and initiate the expression of downstream genes [146].

Another exciting new tool involves a GPCR-coupled CRISPR-Cas system. This system can sense the activation of GPCRs, through the TANGO or Chacha systems described above. Then, this results in the uncaging of dCas9, which goes on to modulate gene expression [182]. In the future, it will be exciting to include optogenetic activation in this approach, by using for example, iTango or light controlled dCas9. This would allow for an experimenter defined time window when GPCR activation uncages dCas9 and modulates gene expression.

### Molecules, systems and behavior

The incredible success of systems neuroscience is in no small part shaped by the foundational methods of the field, including electrophysiology and modeling. It is absolutely remarkable how far the field has come despite the fact that the majority of studies were focused on one group of phenomena (neuronal firing dynamics) of a single class of cells in the brain (neurons), and they were carried out with different variants of a single experimental approach (electrophysiology). This success is all the more remarkable since we now have overwhelming evidence that the complexity of behavior derives from nearly every cell type in the brain, and can be traced back to a wide range of biological mechanisms that go well beyond neuronal firing dynamics. Nevertheless, the majority of ideas and models in systems neuroscience to this day remain firmly entrenched in the illustrious history of the field, in no small part due to the enormous creativity and generative potential of the ideas that serve as its foundational stones. Despite this, it is now time to look into the future of systems neuroscience, a future that will be firmly grounded within its enormously successful past without being restricted by it. The limited sample of exciting technological developments that we briefly reviewed here suggest that it may be worthwhile to leverage these powerful techniques to reimagine the approaches and questions asked in the field. Like many others in the field, we suspect that attempting to understand systems only in molecular terms would be like studying art by investigating the molecular properties of the materials used. This would not only be shortsighted, but it would once again circumscribe the future of the field in a narrow conceptual space. Our view is that imaging and manipulating the very molecular properties that both shaped brain systems (not just neuronal circuits) during evolution, and that now account for and constrain much of their functionality, will open countless windows into the properties and mechanisms of the systems (neuronal, glial, etc.) that underlie the marvelously perplexing complexity of brain states and behavior.

## Data Availability

Not applicable.

## Abbreviations

ACC: Anterior cingulate cortex
AKAR: A-kinase activity reporter
AMPAR: α-Amino-3-hydroxy-5-methyl-4-isoxazolepropionic acid receptor
AsLOV2: Light-Oxygen-Voltage 2 domain of Avenasativa phototropin 1
BLINK1: Blue-light-induced K^+^ channel 1
BLUF: Blue-light-using flavin adenine dinucleotide
BRET: Bioluminescent resonance energy transfer
CALI: Chromophore-Assisted Laser or light Inactivation
Cdk5: Cyclin Dependent Kinase 5
CIB1: Cryptochrome-interacting basic-helix-loop-helix
CLICR: Clustering indirectly using cryptochrome 2
CMOS: Complementary metal–oxide–semiconductor
cpGFP: Circularly permuted green fluorescent protein
CRAC: Ca^2+^ -release–activated Ca^2+^ channel
CREB: The cAMP-response element binding protein
CRISPR: Clustered regularly interspaced short palindromic repeats
CRY: Cryptochrome
Crz1: Calcineurin-responsive zinc finger 1
dCas: Nuclease-dead Caspase
ddFPs: Dimerization-dependent fluorescent proteins
DrBphP: Deinococcus radiodurans bacterial phytochrome
Erk: Extracellular signal-regulated kinase
FGFR1: Fibroblast growth factor receptor 1
FKBP: FK506-binding protein
Flp: Flippase
FRB: FKBP Rapamycin binding domain
FRET: Fluorescence resonance energy transfer
FRT: Flippase recognition target
GABA: Gamma-aminobutyric acid
GFP: Green Fluorescent Protein
GPCR: G protein-coupled receptor
GRAB: Genetically-encoded GPCR-activation based
GTP: Nucleotide guanosine triphosphate
iGluR: Ionotropic glutamate receptor
iLMO: Inhibitory luminescent opsin
IM-LARIAT: Light-activated reversible inhibition by assembled trap of intracellular membranes
kikGR: Kikume green–red
LAMAs: Ligand-modulated antibody fragments
LED: Light-emitting diode
LID: Light-inducible dimerization
LMO: Luminescent opsin
LOV2: Light-Oxygen-Voltage 2
LOVTRAP: LOV2 trap and release of protein
Loxp: Locus of X-over P1
mGluR: Metabotropic glutamate receptor
NAc: Nucleus accumbens
nAChR: Nicotinic acetylcholine receptor
NES: Nuclear export signals
NLS: Nuclear localization signals
P35: Protein of 35 kDa
P25: Protein of 25 kDa
PA-FP: Photoactivatable fluorescent protein
PA-Rac1: Photoactivatable Rac1
POI: Protein of interest
PhyB: Phytochrome B
Phy: Phytochrome
PIF: Phytochrome-interacting factor
PIP3: Phosphatidylinositol (3,4,5)-trisphosphate
PKA: Protein kinase A
pMag-nMag: Positive magnet- negative magnet
PPI: Protein–protein interactions
Rac1: Ras-related C3 botulinum toxin substrate 1
RAF: Rapidly accelerated fibrosarcoma
RET: Resonance energy transfer
ROS: Reactive oxygen species
sgRNA: Single guide RNA
SNARE: Soluble N-Ethylmaleimide-Sensitive Factor Attachment Protein Receptor
SPARK: Specific Protein Association tool giving transcriptional Readout with rapid Kinetics
STIM1: Stromal interaction molecule
TCS: TEV cleavage site
TEVp: Tobacco Etch Virus nuclear-inclusion-a endopeptidase
TF: Transcription factor
TRE: Tetracycline responsive element
TrkB: Tropomyosin receptor kinase B or Tyrosine receptor kinase B
tTA: Tetracycline-controlled transactivator
TULIPs: Tunable Light-Inducible dimerization tags
UCNPs: Upconversion nanoparticles
UV-B: Ultraviolet-B radiation
UVR8: UV Resistance Locus 8
VAMP2: Vesicle associated membrane protein 2
Zdk: Zdark
